# Kanglaite Combined With Epidermal Growth Factor Receptor-Tyrosine Kinase Inhibitor Therapy for Stage III/IV Non-Small Cell Lung Cancer: A PRISMA-Compliant Meta-Analysis

**DOI:** 10.3389/fphar.2021.739843

**Published:** 2021-09-13

**Authors:** Fanming Kong, Chaoran Wang, Xiaojiang Li, Yingjie Jia

**Affiliations:** ^1^ Department of Oncology, First Teaching Hospital of Tianjin University of Traditional Chinese Medicine, Tianjin, China; ^2^ National Clinical Research Center for Chinese Medicine Acupuncture and Moxibustion, Tianjin, China; ^3^ Graduate School, Tianjin University of Traditional Chinese Medicine, Tianjin, China

**Keywords:** EGFR-TKI, kanglaite, NSCLC, meta-analysis, stage III/IV

## Abstract

**Objective:** Kanglaite(KLT), a type of Chinese medicine preparation, is considered as an adjuvant therapeutic option for malignant cancer treatment. This study aimed to systematically investigate the efficacy and safety of the combination of KLT and epidermal growth factor receptor-tyrosine kinase inhibitor (EGFR-TKI) for the treatment of stage III/IV non-small cell lung cancer.

**Methods:** Randomized controlled trials (RCTs) that compared KLT plus EGFR-TKI with EGFR-TKI alone for the treatment of stage III/IV non-small cell lung cancer were reviewed. Literature searches (up to July 10, 2021) were performed on PubMed, Web of Science, Cochrane Library, Embase, ClinicalTrials.gov, China National Knowledge Infrastructure (CNKI), Wanfang Database, and the Chinese Scientific Journal Database. Two researchers independently assessed the risk of bias with the tool of Cochrane Collaboration. RevMan 5.3.0 was used in the analysis of the included trial data.

**Results:** 12 RCTs recruiting 1,046 patients with stage III/IV NSCLC were included. Results showed that compared with EGFR-TKI alone, KLT plus EGFR-TKI significantly increased the disease control rate (DCR) (odds ratio [OR]=3.26; 95% confidence interval [CI]:2.22–4.77; *p* < 0.00001), the objective response rate (ORR) (OR=2.59; 95% CI:1.87–3.58; *p* < 0.00001) and Karnofsky performance status (KPS) (OR = 2.76; 95% CI:1.73–4.39; *p* < 0.00001). Furthermore, patient immunity was enhanced with KLT plus EGFR-TKI. The combined treatment increased the percentage of CD4 + T cells (weighted mean difference [WMD]=5.36; 95% CI:3.60–7.13; *p* < 0.00001),the CD4+/CD8 + ratio (WMD = 0.18; 95% CI: 0.08–0.27; *p* = 0.004), and percentage of NK cells (WMD=4.84; 95% CI: 3.66–6.02; *p* < 0.00001).With regard to drug toxicity, the occurrence rate of nausea and vomiting was significantly reduced by KLT plus EGFR-TKI (OR=0.37; 95% CI: 0.16–0.86; *p* = 0.02).

**Conclusion:** KLT plus EGFR-TKI was effective in treating stage III/IV non-small cell lung cancer. Thus, its application in these patients is worth promoting. Additional double-blind, well-designed and multicenter RCTs are required to confirm the efficacy and safety of this treatment.

## Introduction

Lung cancer remains the leading cause of cancer-related death worldwide (Sung et al., 2021). Despite the recent remarkable progress in screening, diagnosis, and treatment. 57% of patients with lung cancer are diagnosed at an advanced or metastatic stage, during which the 5-years relative survival rate is only 5% (Richards et al., 2017). Epidermal growth factor receptor-tyrosine kinase inhibitors (EGFR-TKIs) have improved clinical benefits for patients with metastatic non-small cell lung cancer (NSCLC) patients (Rosell et al., 2012). The National Comprehensive Cancer Network NSCLC Panel recommends identification of EGFR mutations for all patients with adenocarcinoma. EGFR-TKI combined with chemotherapy improved progression free survival (PFS) in untreated advanced NSCLC patients with EGFR mutation (Hosomi et al., 2020). However, the undesirable effects of EGFR-TKIs adversely affect the quality of life and treatment compliance of patients (Shah and Shah, 2019). Of patients with EGFR-mutated NSCLC, 20–40% experience primary resistance to first or second-generation EGFR-TKIs, which is attributed to genetic alterations (Wang et al., 2016). Moreover, some patients do not show a good initial clinical response. Therefore, a EGFR-TKI-based combination treatment regimen may be more beneficial.

Traditional Chinese medicine (TCM) has been widely used as an adjuvant therapeutic option for cancer treatment (Xiang et al., 2019;Liu S.-H. et al., 2021). High-level clinical studies of TCM injection in cancer care has gradually increased (Yang et al., 2021). Kanglaite injection had been approved by the Chinese State Food and Drug Administration (SFDA) for the treatment of various malignant tumors. KLT is extracted and isolated from coix seeds (Coix lacryma-jobi). A few clinical studies on KLT for patients with solid tumor have been approved in the United States. KLT is the first TCM to be approved by the US Food and Drug Administration (FDA). The clinical mechanisms of KLT for NSCLC are related to the promotion of cancer cell apoptosis, inhibition of migration and proliferation and improvement of the immunity (Pan et al., 2012; Lu et al., 2013; Luo et al., 2017; Duan, 2018; Wu et al., 2018).

**FIGURE 1:**
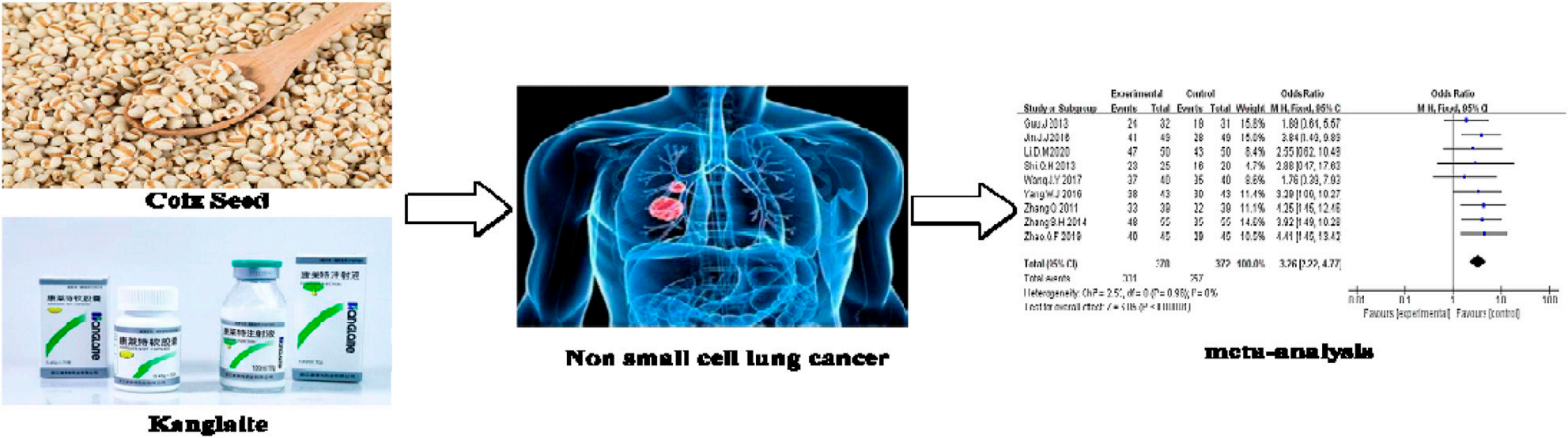
Work flow of the present study.

In previous studies, KLT injection combined with platinum-based chemotherapy showed significantly higher efficacy in the treatment of stage III/IV NSCLC (Hailang et al., 2020a; Li et al., 2020; Ni et al., 2021). With the widespread application of EGFR-TKI, the number of published clinical studies on KLT combined with EGFR-TKI has been increasing. On the basis of previous clinical studies, we performed a PRISMA-compliant meta-analysis of KLT combined with EGFR-TKI in patients with stage III/IV NSCLC (Figure 1) to assess the clinical efficacy, quality of life, immune function (including percentages of CD3^+^, CD4^+^, NK cells and the CD4+/CD8+ratio) and adverse events. This work was conducted to provide comprehensive evidence for further studies and explore the clinical outcome of combination therapy with KLT and EGFR-TKI.

## Materials and Methods

We performed this meta-analysis following the Preferred Reporting Items for Systematic Reviews and Meta-Analyses (PRISMA) guidelines and Cochrane Handbook. The data were obtained from published trials. As this study does not involve animal and patient experiments, the ethical approval was not required.

### Literature Source and Search Strategy

A comprehensive literature search was conducted by two independent researchers (FM Kong and CR Wang). Published studies were retrieved from eight databases including PubMed, Web of Science, Cochrane Library, Embase, ClinicalTrials.gov, the China National Knowledge Infrastructure (CNKI), Wanfang Database and the Chinese Scientific Journal Database. The last search date was July 10, 2021. In addition, we searched the relevant systematic reviews and meta-analyses to find the potential studies that may have been missed in the online searches.

The following search terms were used: “Lung Cancer” or “Non small cell lung cancer” or “NSCLC” or “Lung Carcinoma”or “Carcinoma of the lung” and “Kanglaite” and “Gefitinib” or “Erlotinib” or “Icotinib” or “Afatinib” or “Dacomitinib” or “Osimertinib” or “EGFR-TKI”. No language limits were applied.

### Types of Studies and Selection Criteria

All RCTs that compared KLT plus EGFR-TKI with EGFR-TKI alone were selected and assessed for inclusion in the study.

The inclusion criteria were as follows:

1) Randomized controlled trials (RCTs).
2) Patients with stage III/IV NSCLC confirmed by cytology or pathology.
3) Studies that included >30 patients with NSCLC.
4) Studies that compared the clinical outcomes of EGFR-TKI plus KLT adjuvant therapy (experimental group) with those of EGFR-TKI alone (control group).
5) The EGFR-TKIs included Gefitinib, Icotinib, Erlotinib, Afatinib, Dacomitinib, and Osimertinib.

The exclusion criteria were as follows:

1) Neither RCT nor “random”was mentioned.
2) Articles without sufficient data available.
3) Duplication of previous publications.
4) Case studies, review papers, comments, and conference abstracts.

### Data Extraction and Quality Assessment

Data were independently extracted by two reviewers (FM Kong and CR Wang) according to the above inclusion and exclusion criteria; any disagreement was adjudicated by a third reviewer (XJ Li).

The following data were extracted:

1) The first author’s name
2) Year of publication
3) Study location
4) Tumor stage
5) Number of cases
6) Age of the patients
7) Gender of the patients
8) Therapeutic regimens
9) Main outcomes

### Outcome Definition

The clinical responses assessed included treatment efficacy, performance status, immune function, and adverse events. Treatment efficacy was evaluated in terms of the disease control rate (DCR), objective response rate (ORR), Karnofsky performance status (KPS), immune function indicators (percentages of CD3^+^, CD4^+^, NK cells and the CD4+/CD8+ratio) and adverse events including rash, nausea and vomiting, diarrhea, and liver injury.

### Risk of Bias Assessment

Two researchers (XJ Li and YJ Jia) independently assessed the risk of bias with the tool of Cochrane Collaboration (Higgins et al., 2011). The Cochrane Collaboration contained the following assessment tools: random sequence generation; allocation concealment; blinding of participants, and personnel; blinding of outcome assessment; incomplete data; selective reporting and other bias. Each study was classified as“low risk of bias”,“unclear risk of bias”or“high risk of bias”. Any disagreement was settled through the third researcher (FM Kong).

### Statistical Analysis

Statistical analysis was performed with the RevMan 5.3 software (Nordic Cochran Centre, Copenhagen, Denmark) software. Treatment effects were mainly represented by odds ratio (OR), and continuous data were shown as the weighted mean difference (WMD) with a 95% confidence intervals (CI). *p* value < 0.05 was considered statistically significant. Heterogeneity among the studies was assessed by Cochran’s Q test; I^2^ < 50% or *p* > 0.1 indicated a lack of heterogeneity among the studies. When the level of heterogeneity was small (I^2^< 50%), a fixed-effects model was applied for estimation; otherwise, a random-effects model was selected.

## Results

### Search Results

**FIGURE 2:**
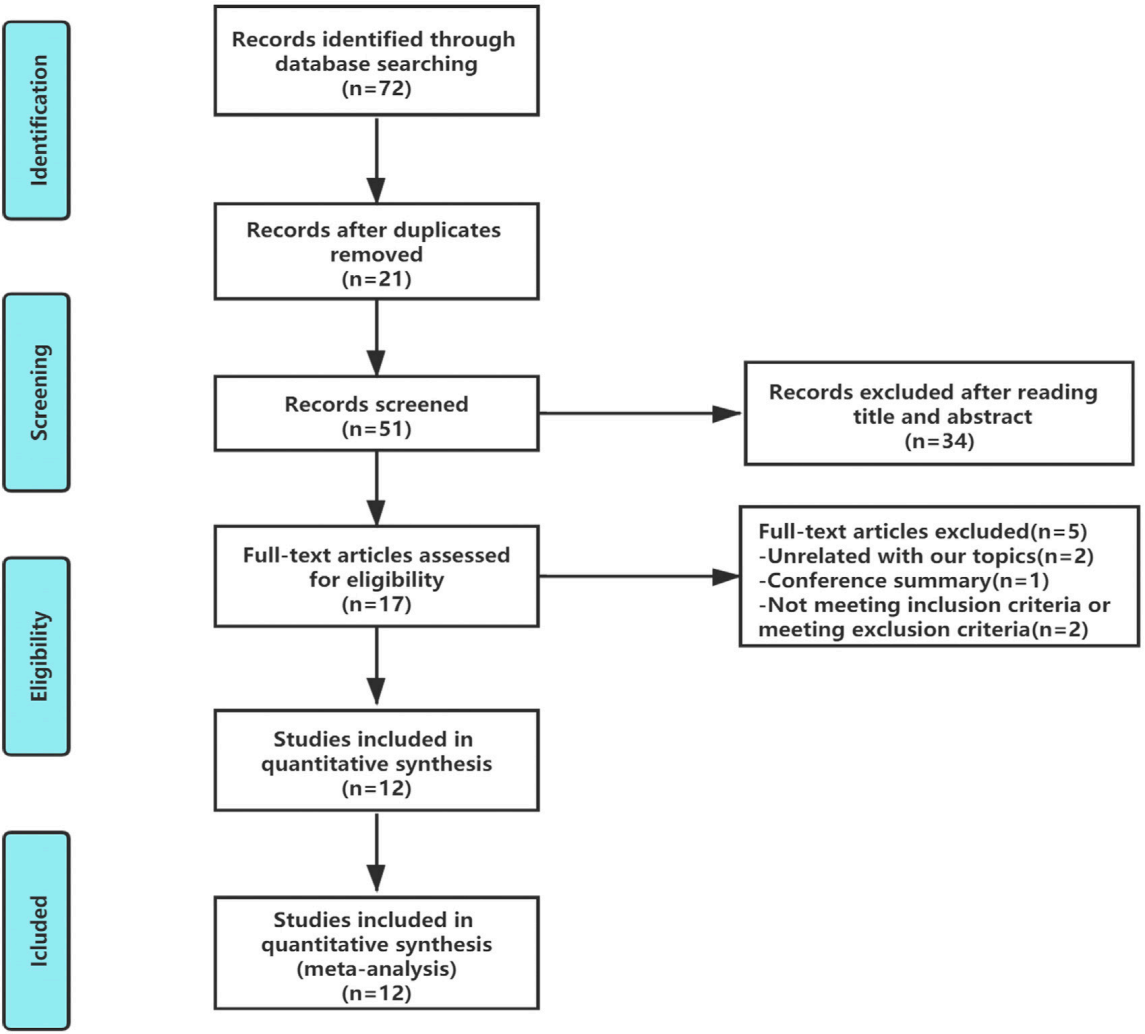
Flow diagram showing study selection for the meta-analysis.

In total, 72 articles were initially identified. Of these articles, 21 papers were excluded because they were duplicates. After the title and abstract review, 34 articles were further excluded. After a detailed assessment of the full text articles, those unrelated with our topics (*n* = 2), conference summary (*n* = 1), and those that did not meeting the inclusion criteria or exclusion criteria (*n* = 2) were also excluded. Ultimately, 12 trials involving a total of 1,046 patients were included in this analysis (Figure 2).

### Characteristics of Included Studies

Table 1 summarizes the basic characteristics of the 12 RCTs. All the studies were conducted in different medical centers in China (Zhang and Yuan, 2011; Guo and Wang, 2013; Shi and Chen, 2013; Zeng et al., 2014; Zhang and Zhou, 2014; Ning et al., 2015; Jin et al., 2016; Yang, 2016; Yang et al., 2016; Wang et al., 2017; Zhao et al., 2019; Li, 2020). A total of 1,046 patients were recruited, of which 526 patients with stage III/IV NSCLC were treated with EGFR-TKI in combination with KLT, while 520 patients were treated with EGFR-TKI alone, with a sample size of 46–186 in each trial. The course of treatment lasted from 3 to 9 weeks. The control group received EGFR-TKIs, including gefitinib (Zhang and Yuan, 2011; Shi and Chen, 2013; Zeng et al., 2014; Zhang and Zhou, 2014; Ning et al., 2015; Jin et al., 2016; Yang et al., 2016; Wang et al., 2017; Zhao et al., 2019; Li, 2020), Icotinib (Yang, 2016) and Erlotinib (Guo and Wang, 2013). The patients in the experimental group received EGFR-TKI in combination with KLT, including KLT injections (Zhang and Yuan, 2011; Guo and Wang, 2013; Shi and Chen, 2013; Zeng et al., 2014; Zhang and Zhou, 2014; Ning et al., 2015; Jin et al., 2016; Yang, 2016; Yang et al., 2016; Zhao et al., 2019) and KLT capsules (Wang et al., 2017; Li, 2020). Patient characteristics are shown in Table 1.

**TABLE 1:** Clinical information from the eligible trials included in the meta-analysis.

Included studies	TNM stage	Patients Exp/Con	Therapeutic regimen		Dosage of kanglaite (daily)	Dose of EGFR-TKI (daily) (mg)	Duration	Outcomes measure
			Experimental	Control				
Ning.J.L (2015)	ⅢB-Ⅳ	93/93	Con + KLT^a^	Gefitinib	200 ml	250	8 weeks	IF
Zhang.S.H (2014)	Ⅲ-Ⅳ	55/55	Con + KLT^a^	Gefitinib	200 ml	250	9 weeks	DCR ORR KPS AE
Li.D.M (2020)	ⅢB-Ⅳ	50/50	Con + KLT^b^	Gefitinib	2.7 g Qid	250	Not provide	DCR ORR AE
Wang.J.Y (2017)	ⅢB-Ⅳ	40/40	Con + KLT^b^	Gefitinib	2.7 g Qid	250	60 d	DCR ORR AE
Zeng.H.X (2014)	ⅢB-Ⅳ	23/23	Con + KLT^a^	Gefitinib	200 ml	250	6 weeks	IF AE KPS
Yang.L (2016)	Ⅲ-Ⅳ	32/32	Con + KLT^a^	Gefitinib	200 ml	250	6 weeks	IF
Yang.W.J (2016)	ⅢB-Ⅳ	43/43	Con + KLT^a^	Icotinib	200 ml	375	9 weeks	DCR ORR KPS IF AE
Guo.J (2013)	ⅢB-Ⅳ	32/31	Con + KLT^a^	Erlotinib	200 ml	150	9 weeks	DCR ORR IF AE
Jin.J.J (2016)	Ⅲ-Ⅳ	49/49	Con + KLT^a^	Gefitinib	200 ml	250	3 weeks	DCR ORR IF AE
Zhang.Q (2011)	Ⅲ-Ⅳ	39/39	Con + KLT^a^	Gefitinib	100 ml	250	9 weeks	DCR ORR KPS AE
Zhao.Q.F (2019)	Ⅲ-Ⅳ	45/45	Con + KLT^a^	Gefitinib	200 ml	251	3 weeks	DCR ORR IF AE
Shi.Q.H (2013)	ⅢB-Ⅳ	25/20	Con + KLT^a^	Gefitinib	100 ml	250	60 d	DCR ORR KPS

Con, control group; Exp, experimental group; DCR, disease control rate; ORR, objective response rate; KPS, Karnofsky performance status; IF, Immune function; AE, adverse event.

a Kanglaite injection.

b Kanglaite capsule.

### Risk of Bias Assessment

**FIGURE 3:**
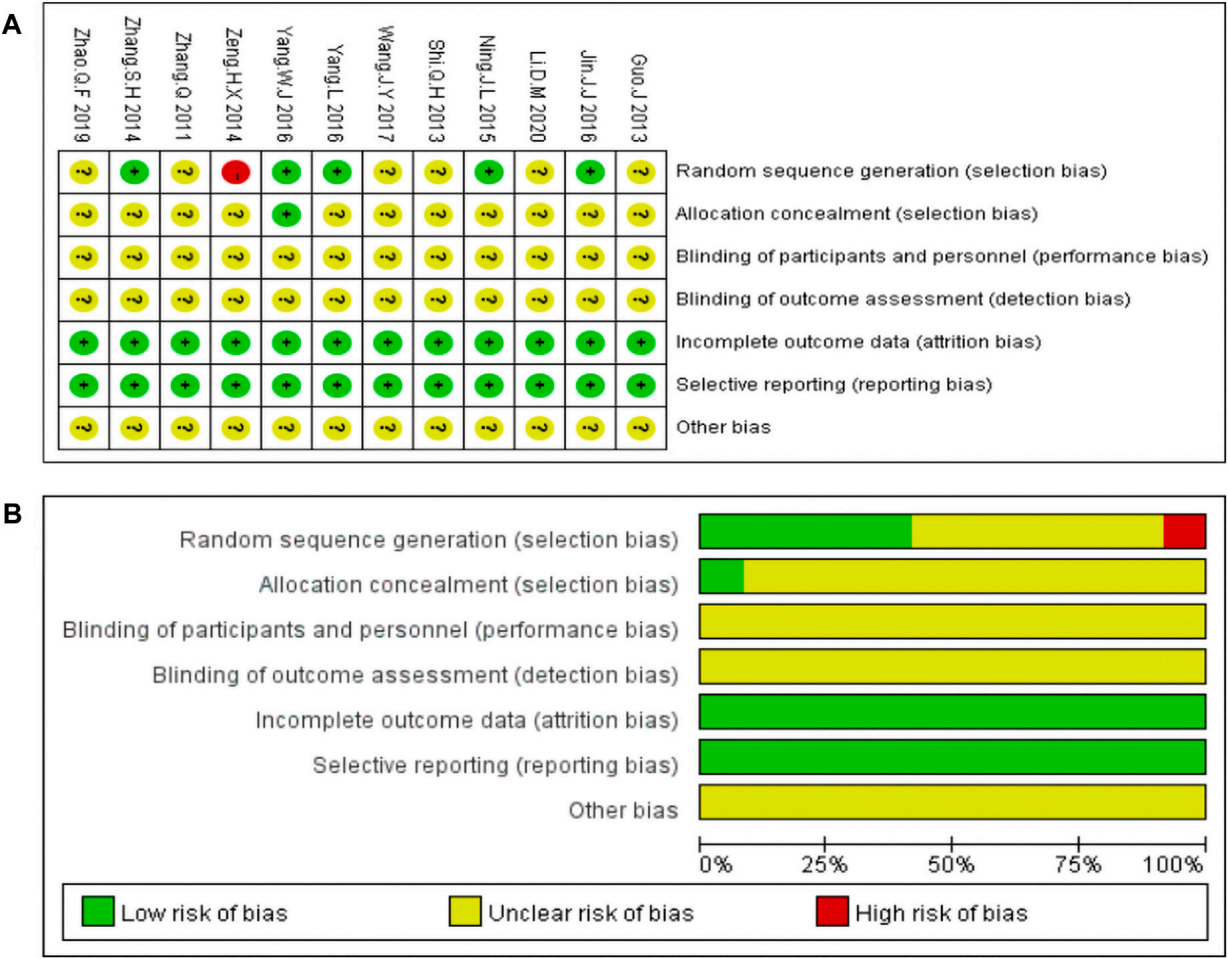
Risk of bias of the included studies **(A)** Risk of bias graph **(B)** Risk of bias summary.

The bias risk analysis performed using the Cochrane Collaboration tool revealed bias in all the included studies. “Random” or “randomized” or “randomization” was mentioned in 11 studies, except for one study (Zeng et al., 2014). Only one study reported allocation concealment (Yang, 2016). All the included studies reported detailed outcome data. None of the 12studies provided blinding of the outcome data, clear descriptions of selective and other biases. Risk of bias assessment are shown in Figure 3.

### Outcomes Measures

#### Disease Control Rate and Objective Response Rate

**FIGURE 4:**
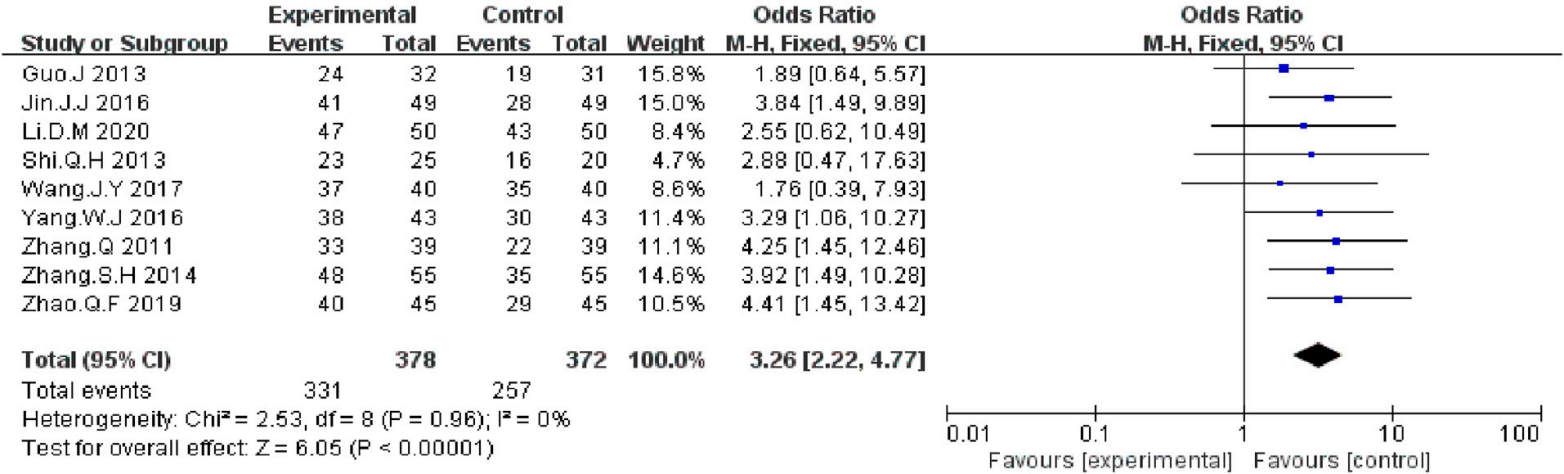
Forest plot of DCR in patients treated with KLT + EGFR-TKI and EGFR-TKI alone.

**FIGURE 5:**
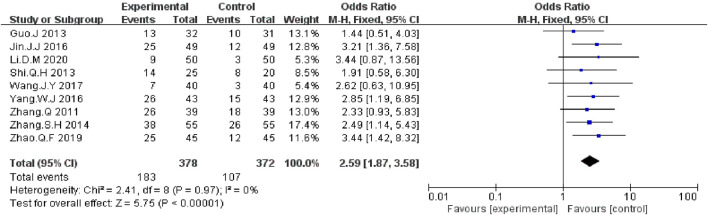
Forest plot of ORR in patients treated with KLT + EGFR-TKI and EGFR-TKI alone.

We extracted the data on DCR and ORR data from nine included RCTs report (Zhang and Yuan, 2011; Guo and Wang, 2013; Shi and Chen, 2013; Zhang and Zhou, 2014; Jin et al., 2016; Yang, 2016; Wang et al., 2017; Zhao et al., 2019; Li, 2020). The meta-analysis data showed that, compared with EGFR-TKI alone, KLT plus EGFR-TKI significantly improved DCR (OR = 3.26; 95% CI:2.22–4.77; *p* < 0.00001) (Figure 4) and ORR (OR: 2.59; 95% CI: 1.87–3.58; *p* < 0.00001) (Figure 5). Statistical homogeneity was observed for both outcomes (I^2^ = 0%).

#### Karnofsky Performance Status

**FIGURE 6:**
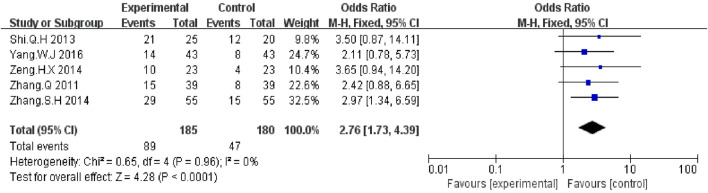
Forest plot of KPS improvement in patients treated with KLT + EGFR-TKI and EGFR-TKI alone.

Five trials (Zhang and Yuan, 2011; Shi and Chen, 2013; Zeng et al., 2014; Zhang and Zhou, 2014; Yang, 2016) with a total of 750 patients reported improvement in KPS improvement. According to the results, the KPS was significantly higher in the KLT plus EGFR-TKI group than the control group (OR = 2.76, 95% CI:1.73–4.39; *p* < 0.00001) (Figure 6). A fixed-effect model was used due to the heterogeneity (I^2^ = 0%).

#### Immune Function

**FIGURE 7:**
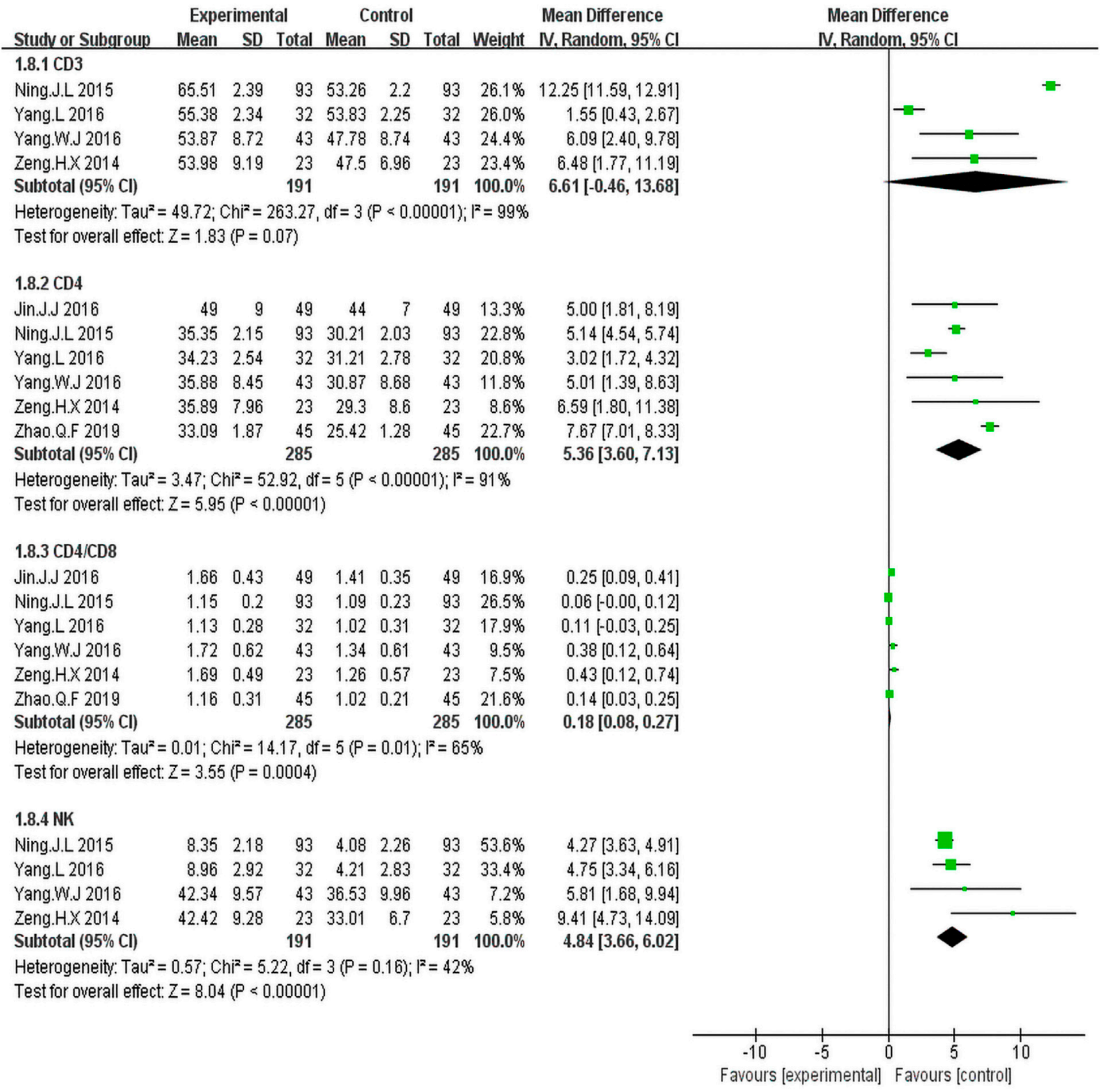
Forest plot of Immune function in patients treated with KLT + EGFR-TKI and EGFR-TKI alone.

Four trials (Zeng et al., 2014; Ning et al., 2015; Yang, 2016; Yang et al., 2016) including 382 patients reported percentages of CD3^+^ cells. Statistical heterogeneity was observed (I^2^ = 99%, *p* < 0.00001); Hence, the random effect model was employed. The results showed that the percentage of CD3^+^ cells was similar between the KLT plus EGFR-TKI group and control group (WMD = 6.61; 95% CI: 0.46 to 13.68; *p* = 0.07) (Figure 7).

Six trials (Zeng et al., 2014; Ning et al., 2015; Jin et al., 2016; Yang, 2016; Yang et al., 2016; Zhao et al., 2019) including 570 patients reported percentage of CD4+cells and the CD4+/CD8+ratio.There was statistical heterogeneity. The random effect model was employed for the analysis. The results illustrated that KLT plus EGFR-TKI group had an advantage of increased percentage of CD4+cells (WMD = 5.36; 95%CI: 3.60–7.13; *p* < 0.00001; I^2^ = 91%) and the CD4+/CD8+ ratio (WMD = 0.18; 95% CI: 0.08–0.27; *p* = 0.004; I^2^ = 65%) (Figure 7).

Four trials (Zeng et al., 2014; Ning et al., 2015; Yang, 2016; Yang et al., 2016) including 382 patients reported percentage of NK cells. The results showed that the KLT plus EGFR-TKI group had an advantage of increased NK cells (WMD = 4.84; 95% CI: 3.66–6.02; *p* < 0.00001; I^2^ = 42%) (Figure 7).

#### Adverse Event

**FIGURE 8:**
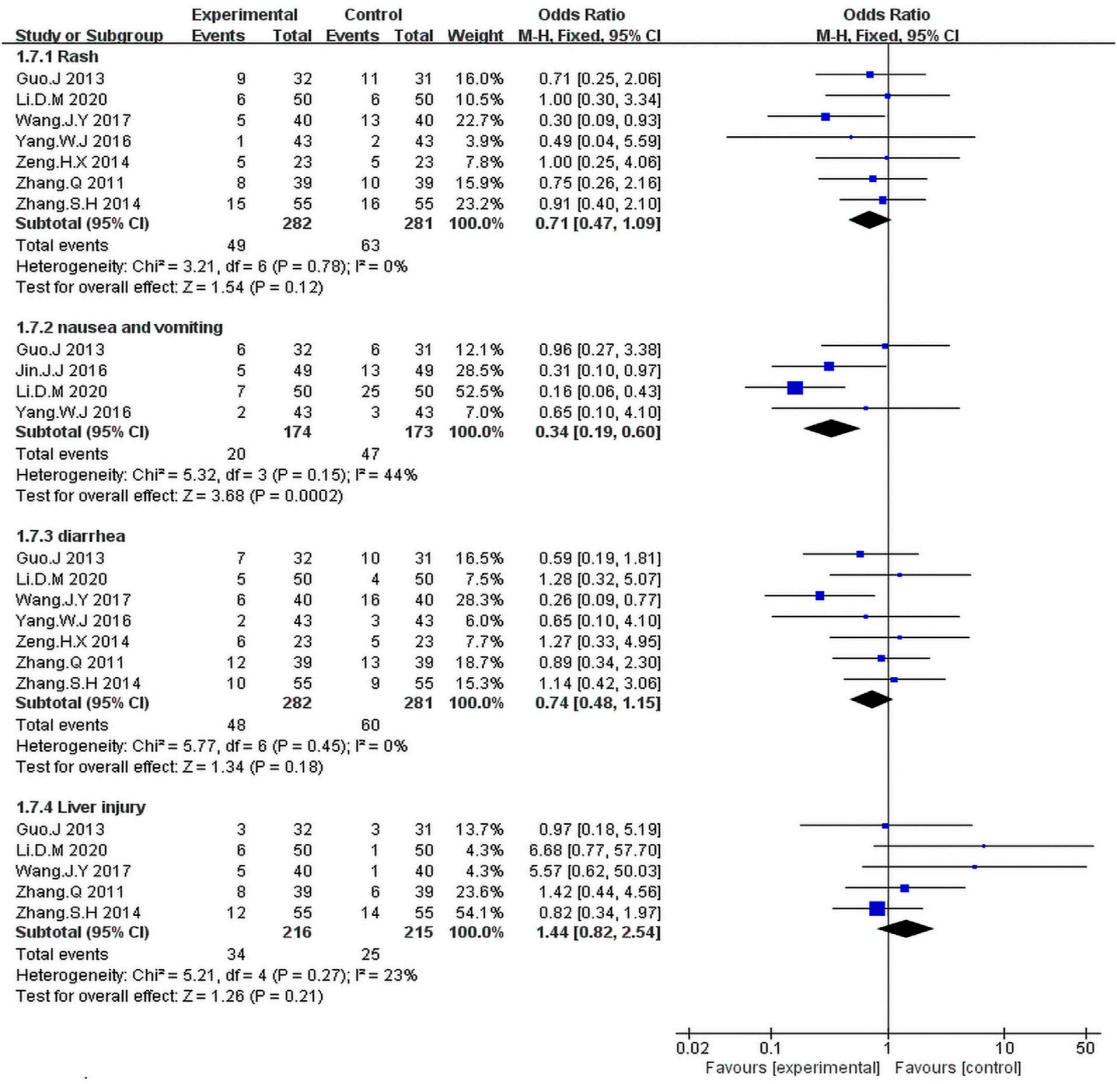
Forest plot of adverse events in the patients treated with EGFR-TKI + KLT and EGFR-TKI alone.

The fixed-effects meta-analysis revealed a significantly lower occurrence rate of nausea and vomiting in the KLT plus EGFR-TKI group than in the control group (OR = 0.37; 95% CI:0.16–0.86; *p* = 0.02; I^2^ = 44%) (Figure 8).

We identified seven studies (Zhang and Yuan, 2011; Guo and Wang, 2013; Zeng et al., 2014; Zhang and Zhou, 2014; Yang, 2016; Yang et al., 2016; Wang et al., 2017) including 563 patients with rash and diarrhea. The Fixed-effects model revealed that the KLT plus EGFR-TKI group showed no significant differences in the occurrence rates of rash (OR = 0.71; 95% CI:0.47–1.09; *p* = 0.12; I^2^ = 0%) and diarrhea (OR = 0.74; 95% CI:0.48–1.15; *p* = 0.18; I^2^ = 0%) compared with the control group (Figure 8).

As can be seen in Figure 8, five studies (Zhang and Yuan, 2011; Guo and Wang, 2013; Zhang and Zhou, 2014; Wang et al., 2017; Li, 2020) including 431 patients reported the incidence of liver injury. The meta-analysis revealed that the combination treatment with KLT plus EGFR-TKI did not significantly reduce the risk of liver injury risk when compared with the EGFR-TKI alone (OR = 1.44; 95% CI: 0.82–2.54; *p* = 0.21; I^2^ = 23%).

#### Sensitivity Analysis

We performed a subgroup analysis to examine the source of the heterogeneities in ORR and DCR with regard to sample size, form of KLT, duration of therapy. As shown in Table 2, there were no significant differences between sample size, form of KLT, and therapy duration. Moreover, in terms of DCR index, KLT injection plus EGFR-TKI was found to be more effective than KLT capsule plus EGFR-TKI.

**TABLE 2:** Subgroup analyses of DCR and ORR between the experimental and control group.

Parameter	Factors at study	Experimental Patients(n)	Control Patients(n)	Analysis method	I^2^	Odds ratio (OR)	95%CI	*p*-value
**DCR**	**Sample size**							
	≥80	282	282	Fixed	0	3.44	2.17–5.44	< 0.00001
	< 80	96	90	Fixed	0	2.87	1.43–5.74	0.003
	**Form of KLT**							
	Injection	288	282	Fixed	0	3.48	2.31–5.46	< 0.00001
	Capsule	90	90	Fixed	0	2.15	0.77–6.01	0.14
	**Duration**							
	3 W	94	94	Fixed	0	1.42	1.19–1.70	0.0001
	9 W	169	168	Fixed	0	1.34	1.18–1.53	< 0.00001
**ORR**	**Sample size**							
	≥80	282	282	Fixed	0	2.96	2.01–4.37	< 0.00001
	< 80	96	90	Fixed	0	1.89	1.04–3.42	0.04
	**Form of KLT**							
	Injection	288	282	Fixed	0	2.54	1.80–3.58	< 0.00001
	Capsule	90	90	Fixed	0	3.03	1.13–8.14	0.003
	**Duration**							
	3 W	94	94	Fixed	0	3.32	1.79–6.15	0.0001
	9 W	169	168	Fixed	0	2.29	1.47–3.57	0.0002

#### Publication Bias

**FIGURE 9:**
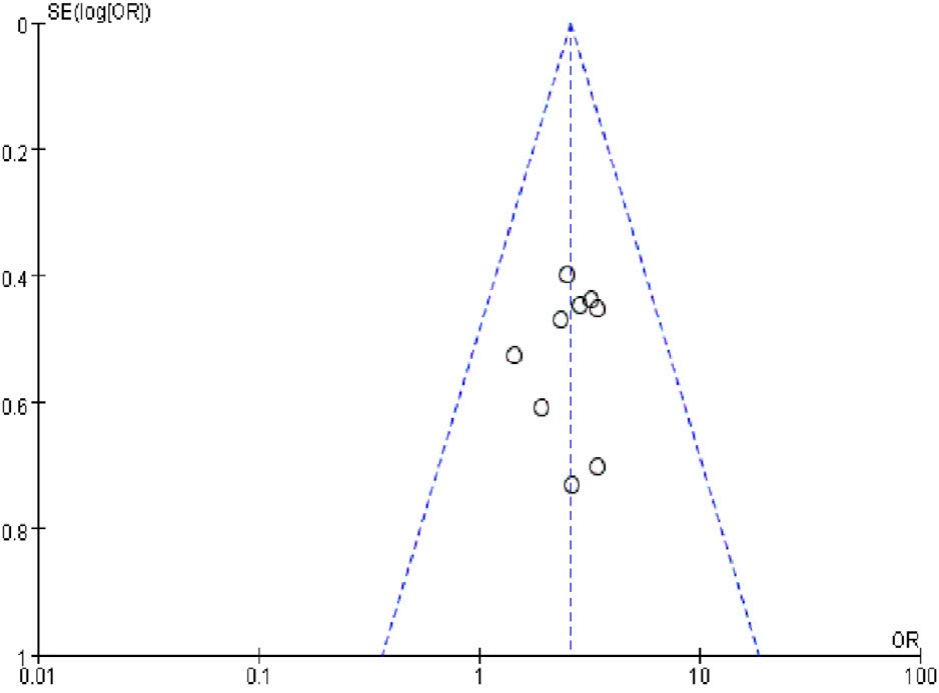
Funnel plot of publication bias.

A funnel plot of the nine studies that reported ORR data was used to assess publication bias (Figure 9). The funnel plot was asymmetrical, indicating the existence of publication bias.

## Discussion

Clinically, KLT has synergistic effects with radiotherapy and chemotherapy, it has been used in the adjuvant treatment of various tumors such as lung cancer (Hailang et al., 2020a; Li et al., 2020), breast Cancer (Liu S. et al., 2021), hepatocellular carcinoma (Liu et al., 2019; Dou et al., 2020)and colorectal cancer (Mao et al., 2020). The main effects targeted the improvements of clinical efficacy, performance status, KPS score, immune function and the reduction of the side effects of radiotherapy and chemotherapy. In advanced NSCLC, KLT injection combined with platinum-based chemotherapy showed significantly higher efficacy (Wen et al., 2020). EGFR-TKIs have proved effective as first-or second-line therapy for advanced NSCLC. Although the achieved efficacy is significant, most patients acquire resistance to the EGFR-TKIs, which limits the benefits of this treatment. Chinese herbal extracts play a role in overcoming EGFR-TKI resistance in NSCLC (Lee et al., 2021). TCM combined with EGFR-TKI treatment prolonged progression-free survival (PFS) in patients with NSCLC who were harboring EGFR mutations and caused no adverse effects (Jiao et al., 2019; Tang et al., 2019). TCMs have better effects on ORR than EGFR-TKI alone in the treatment of NSCLC (Sui et al., 2020). A systematic review revealed that combination of KLT injection with gefitinib may enhance the therapeutic effectiveness for the patients with NSCLC (Hailang et al., 2020b). However, this study only included seven RCTs up to 2016. The meta-analysis did not include other EGFR-TKIs and different forms of KLT. In recent years, the number of published clinical studies on KLT combined with EGFR-TKI has been increasing. Based on this, we comprehensively searched the literature (up to July 10, 2021) for studies that included icotinib, erlotinib and the capsule form of KLT to systematically evaluate the efficacy and safety of the treatment for stage III/IV non-small cell lung cancer. DCR is regarded as one of the best indicators for predicting OS and PFS (Claret et al., 2014). Our study included DCR as the main outcome indicator. Compared with previous studies, we followed the PRISMA guidelines and additionally performed a subgroup analysis, risk-of-bias assessment, and publication bias analysis for a more comprehensive research.

The meta-analysis was performed with 12 RCTs to evaluate the clinical efficacy of the addition of KLT to EGFR-TKI. The study results showed that compared with EGFR-TKI alone, the combination of KLT and EGFR-TKI significantly improved DCR, ORR, KPS and immune function. The percentages of CD4^+^ cells, NK cells and the CD4+/CD8+ratio were significantly increased when KLT was administered, indicating that the immune function of NSCLC patients was improved. Previous studies have shown that KLT has pronounced immunostimulatory activities in C57BL/6 mice (Pan et al., 2012). KLT in combination with chemotherapy influences the peripheral blood T lymphocyte subsets and blood immunoglobulins in patients with advanced NSCLC (Wen et al., 2020). In the subgroup analysis, no significant differences in ORR and DCR were found between sample size, form of KLT, and therapy duration. This suggests that the dosage form of KLT should be chosen depending on the patient’s condition. Moreover, in terms of DCR index, KLT injection plus EGFR-TKI was found to be more effective than KLT capsule plus EGFR-TKI. The subgroup analysis revealed that even with the increased treatment duration (from 3 to 9 weeks), DCR was not improved, indicating that 3 weeks of KLT therapy might be an optimal choice. All these results indicate that the addition of KLT might enhance the curative effects of treatment for stage III/IV non-small cell lung cancer.

Although the combination of KLT and gefitinib has shown better clinical efficacy, the synergistic mechanisms are currently unclear. Previous basic experiments showed that the clinical mechanisms of action of KLT for lung cancer are related to the promotion of cancer cell apoptosis (Lu et al., 2013), improvement of the immunity (Pan et al., 2012; Duan, 2018), inhibition of migration and invasion (Luo et al., 2017; Wang and Wang, 2019) and inhibition of proliferation (Wu et al., 2018). (Table 3). KLT combined with Gefitinib can reduce the number of angiogenesis in Lewis lung cancer tissues. The vascular endothelial growth factor-kinase domain receptor (VEGF-KDR) pathway may be one of the mechanisms to inhibit tumor angiogenesis. This may be one of the reasons for the synergistic effect in the treatment of lung cancer (Shen et al., 2013). The combination of KLT and gefitinib can induce cell apoptosis significantly. KLT may increase the sensitivity of the human lung adenocarcinoma cell line A549 to gefitinib, (Zhao et al., 2015). At present, the synergistic mechanism of KLT and EGFR-TKI is still unclear, and more experimental data are needed to clarify this.

**TABLE 3:** Main Anticancer Mechanism of Coix seed in treatment of lung cancer.

Pharmacological effects	Types	Main anticancer mechanism	Fraction
Promotes tumor cell apoptosis	NSCL A549 cells	Mechanism of the intrinsic mitochondrial pathway (Lu et al., 2013)	Polysaccharide
Improves the immunity	Lewis lung carcinoma	Regulates the expression of NF-κB/IκB, increases IL-2 (Pan et al., 2012)	Coix seed extract
	Lewis lung carcinoma	Decreases the TAM levels and improves hypoxia status (Duan, 2018)	Coix seed extract
Inhibits migration and invasion	NSCL A549 cells	Inhibits JAK2/STAT3 signaling pathway (Wang and Wang, 2019)	Coix seed extract
	NSCL A549 cells	Downregulates the S100A4 (Luo et al., 2017)	Polysaccharide
Inhibits proliferation	Serum sample	Reduces the expressions of miRNA-21 (Wu et al., 2018)	Coix seed extract

There were some limitations in this study. First, all the trials were published in China, which is a source of selection bias. Only one study on KLT treatment for lung cancer in the United States is registered at ClinicalTrials.gov (NCT01640730). Second, the studies included in this meta-analysis used an“A + B vs. B”design, without a rigorous control for placebo effect. Third, none of the trials reported the OS and PFS rates. Whether KLT plus EGFR-TKI improve the OS and PFS for NSCLC remains unclear. Fourth, only first-generation EGFR-TKIs were used in the studies included in this meta-analysis. No relevant clinical study has used KLT combined with afatinib or osimertinib. Furthermore, our current study has not been registered, and bias may have been present. Future trials are needed to ensure that the reporting follows the consolidated standards for reporting trials guidelines. Additional double-blind, well-designed and multicenter RCTs are required to confirm the efficacy and safety of this combination treatment.

## Conclusion

The results of this study indicate that treatment with KLT combined with EGFR-TKI is more effective than EGFR-TKI alone in the treatment for stage III/IV non-small cell lung cancer patients. KLT can be used as a complementary therapy for NSCLC. However, the low quality of some of the included publications increased the risk of bias, which, to some extent, affected the reliability of the research. The clinical efficacy of KLT-mediated adjuvant therapy for stage III/IV non-small cell lung cancer requires verification in methodologically rigorous trials.

## Data Availability

The original contributions presented in the study are included in the article/supplementary material, further inquiries can be directed to the corresponding author.
